# Decreasing of p27^Kip1^ and cyclin E protein levels is associated with progression from superficial into invasive bladder cancer

**DOI:** 10.1054/bjoc.2000.1736

**Published:** 2001-05

**Authors:** T Kamai, K Takagi, H Asami, Y Ito, H Oshima, K-I Yoshida

**Affiliations:** Department of Urology, Dokkyo University School of Medicine, Tochigi, Japan, Department ofUrology and Pathology, Tokyo Metropolitan Tama Geriatric Hospital, Tokyo, Japan, Department of Urology, Tokyo Medical and Dental University School of Medicine, Tokyo, Japan

**Keywords:** bladder, neoplasm, cell cycle, p27^Kip1^, cyclin E, Ki-67

## Abstract

The p27^Kip1^(p27) protein is a cyclin-dependent kinase inhibitor of the transition from G1 to S phase. It has been reported that decreased p27 protein level is a negative prognostic indicator in human tumours including bladder cancer. We studied the relationship between protein levels of p27, cyclin E and Ki-67 and clinicopathological features of 145 consecutive Japanese patients with transitional cell carcinoma of the bladder using immunohistochemical staining. Low protein levels of p27 were associated with low staining of cyclin E (*P* = 0.0302), high Ki-67 index (*P* = 0.0306), poorly differentiated grade (*P* = 0.0006), muscle invasion (*P* = 0.0019) and lymph node metastsis (*P* = 0.0002). Low staining of cyclin E and high Ki-67 index correlated with poorly differentiated grade, muscle invasion and lymph node metastsis. Cyclin E protein levels was inversely related with Ki-67 index (*P* = 0.0002). Kaplan–Meier plots of survival rate in patients with low versus high p27 staining showed that low protein levels of p27 were associated with a shortened disease-free and overall survival (*P*< 0.0001 and *P*< 0.0001, respectively). Similarly, low staining of cyclin E and high Ki-67 index correlated with a shortened disease-free and overall survival. On multivariate analysis using Cox proportional hazards model, low protein levels of p27 and high Ki-67 index were independent predictors of shortened disease-free (*P*< 0.0001, *P* = 0.0031, respectively), and low protein levels of p27, low staining of cyclin E and high Ki-67 index of overall survival (*P* = 0.0017, *P* = 0.0009, *P* = 0.0003, respectively). In superficial bladder tumours (Ta, T1; 86 patients), significant correlations were observed between low p27 staining and high Ki-67 index and early recurrence (*P* = 0.0048, *P* = 0.0178, respectively). Among the recurrenced superficial tumours (35 patients), the tumours which remained at a low stage showed high protein levels of p27 and cyclin E, and the tumours which progressed to invasive disease showed a gradual decrease in p27 and cyclin E protein levels over time. Our findings suggest that decreased protein levels of p27 and cyclin E play a role in the progression of bladder cancer and to evaluate these protein levels may be useful in management of the diseases. © 2001 Cancer Research Campaign http://www.bjcancer.com


*British Journal of Cancer* (2001) **84**(9), 1242–1251
© 2001 Cancer Research Campaign
doi: 10.1054/ bjoc.2001.1736, available online at http://www.idealibrary.com on IDEAL®

### Decreasing of p27^Kip1 and cyclin E protein levels is associated with progression from superficial into invasive bladder cancer

**T Kamai[1,2], K Takagi[2], H Asami[3], Y Ito[3], H Oshima[4] and K-I Yoshida[1]**

[1]Department of Urology, Dokkyo University School of Medicine, Tochigi, Japan, Department of [2]Urology and [3]Pathology, Tokyo Metropolitan Tama Geriatric Hospital, Tokyo, Japan, [4]Department of Urology, Tokyo Medical and Dental University School of Medicine, Tokyo, Japan

**Summary** The $p27^{Kip1}$ (p27) protein is a cyclin-dependent kinase inhibitor of the transition from G1 to S phase. It has been reported that decreased p27 protein level is a negative prognostic indicator in human tumours including bladder cancer. We studied the relationship between protein levels of p27, cyclin E and Ki-67 and clinicopathological features of 145 consecutive Japanese patients with transitional cell carcinoma of the bladder using immunohistochemical staining. Low protein levels of p27 were associated with low staining of cyclin E ($P = 0.0302$), high Ki-67 index ($P = 0.0306$), poorly differentiated grade ($P = 0.0006$), muscle invasion ($P = 0.0019$) and lymph node metastsis ($P = 0.0002$). Low staining of cyclin E and high Ki-67 index correlated with poorly differentiated grade, muscle invasion and lymph node metastsis. Cyclin E protein levels was inversely related with Ki-67 index ($P = 0.0002$). Kaplan–Meier plots of survival rate in patients with low versus high p27 staining showed that low protein levels of p27 were associated with a shortened disease-free and overall survival ($P < 0.0001$ and $P < 0.0001$, respectively). Similarly, low staining of cyclin E and high Ki-67 index correlated with a shortened disease-free and overall survival. On multivariate analysis using Cox proportional hazards model, low protein levels of p27 and high Ki-67 index were independent predictors of shortened disease-free ($P < 0.0001$, $P = 0.0031$, respectively), and low protein levels of p27, low staining of cyclin E and high Ki-67 index of overall survival ($P = 0.0017$, $P = 0.0009$, $P = 0.0003$, respectively). In superficial bladder tumours (Ta, T1; 86 patients), significant correlations were observed between low p27 staining and high Ki-67 index and early recurrence ($P = 0.0048$, $P = 0.0178$, respectively). Among the recurrenced superficial tumours (35 patients), the tumours which remained at a low stage showed high protein levels of p27 and cyclin E, and the tumours which progressed to invasive disease showed a gradual decrease in p27 and cyclin E protein levels over time. Our findings suggest that decreased protein levels of p27 and cyclin E play a role in the progression of bladder cancer and to evaluate these protein levels may be useful in management of the diseases. © 2001 Cancer Research Campaign http://www.bjcancer.com

**Keywords**: bladder; neoplasm; cell cycle; $p27^{Kip1}$; cyclin E; Ki-67

A general problem in the evaluation of superficial bladder tumours (Ta, T1) is the tendency to underdiagnose the extent of the disease. Treatment decisions for individual patients with superficial bladder tumour have been based on tumour grade. Although intravesical chemotherapy and Bacillus Calmette-Guerin (BCG) immunotherapy reduce disease in superficial bladder tumours (Vegt et al, 1995), intravesical therapies fail to influence the long-term course of superficial disease (Lamm et al, 1995). Furthermore, it has been well known that the majority of superficial bladder tumours recur, and a certain percentage of these will be at a higher stage and progress (Loening et al, 1980; Heney et al, 1983). On the other hand, systemic chemotherapy using methotrexate, vinblastine, adriamycin and cisplatinum (M-VAC) has become widespread as a promising regimen for invasive bladder tumours because of its improved effectiveness over previous chemotherapeutic regimens (Sternberg et al, 1985; Geller et al, 1991). Nevertheless, the prognosis is still poorer than might be expected (Conner et al, 1990). Therefore, elucidation of the underlying molecular mechanisms for recurrence and progression from superficial to invasive disease may offer new treatment strategies.

The malignant transformation of the tumour cell is determined by a sequence of genetic alterations, in which alterations of cell cycle-regulating genes are evolving as important factors of promoting tumour development (Hunter and Pines, 1994). In the mammalian cell cycle, the G1/S restriction point is thought to be the most important checkpoint. $p27^{Kip1}$ (p27) is a negative cell cycle-regulating gene that belongs to the $p21^{WAF/CIP1}$ cyclin-dependent kinase inhibitor family. p27 redistributes from the cyclin D1-Cdk4/6 complex of mid G1 to the cyclin E-Cdk2 complex regulating late G1 (Toyoshima and Hunter, 1994). p27 plays a central role in the transition from late G1 to *S* phase and activation of the cyclin E-Cdk2 complex is the rate-limiting event for transition into *S* phase (Steeg and Abrams, 1997).

Regulation of p27 occurs at the post-translational level. Although mutations in the p27 gene are rare in human cancers, decreased p27 protein levels have a negative prognostic impact in breast (Catzavelos et al, 1997; Porter et al, 1997), lung (Esposito et al, 1997), gastric (Mori et al, 1997), colorectal (Loda et al, 1997), ovarian (Masciullo et al, 1999) and prostate cancer (Tsihlias et al, 1998; Yang et al, 1998; Kuczyk et al, 1999). We

*Received 10 August 2000*
*Revised 2 January 2001*
*Accepted 11 January 2001*

*Correspondence to:* T Kamai have previously reported that low p27 protein levels and a high Ki-67 index were associated with unfavourable prognosis in upper urinary tract cancer (Kamai et al, 2000). Increased expression of cyclin E is associated with shortened survival in breast cancer (Keyomarsi et al, 1997; Porter et al, 1997). In bladder cancer, reduced protein levels of p27 and cyclin E correlate with early recurrence and shortened survival (Del Pizzo et al, 1999; Lee et al, 1999; Sgambato et al, 1999).

To date, there are no available data regarding alterations in these protein levels during the progression from superficial to invasive bladder cancer. Therefore, additional studies are required to elucidate the relationship between p27 and other proteins implicated in G1/S transition (e.g. cyclin E) and in S-phase (e.g. Ki-67) in development of the bladder cancer. At first, we compared protein levels of p27, cyclin E and Ki-67 in tumour cells using immunohistochemical staining with the clinicopathological features of the patients to assess their role in predicting survival. We also analysed these protein levels at recurrence and with progression from superficial to invasive disease to examine the biological behaviour of bladder cancer. We discuss the implications of the findings.

### MATERIALS AND METHODS

### Patients

Biopsy specimens of bladder cancers obtained between 1987 and 1997 from 145 consecutive Japanese patients (101 men, 44 women), 54 to 97 years old (mean age 76.1 years), with newly diagnosed primary transitional cell carcinoma (TCC) of the bladder were examined. All patients routinely had imaging studies (CT and/or MRI) prior to surgery for standard staging. Follow-up ranged from 3 to 124 months (median follow-up, 50 months). Surgical specimens were obtained at the first operation before receiving any therapy. Some patients received adjuvant therapies after the first operation. Generally, intravesical mitomycin C, doxorubicin, and/or BCG was used in superficial tumours after the initial therapy or during follow-up, or at both times when the tumour was high grade or had a high likelihood of recurrence. M-VAC chemotherapy was used in high-grade invasive tumours. Surgical specimens were fixed in buffered formalin (pH 7.0), embedded in paraffin, sectioned at 5 μm, and stained with haematoxylin and eosin. The grade and stage were classified according to the criteria of the TNM staging system (Sobin et al, 1997).

### Immunostaining

Immunohistochemical staining was performed with anti-p27$^{Kip1}$ monoclonal antibody (Transduction Laboratories, Lexington, KY), anti-cyclin E polyclonal antibody (Santa Cruz Biotechnology, Santa Cruz, CA) and anti-Ki-67 monoclonal antibody, MIB-1 (Medical and Biological Laboratories, Nagoya, Japan) using the immunoperoxidase technique and microwave treatment of tissue sections in citrate buffer as described previously (Catzavelos et al, 1997; Loda et al, 1997; Kamai et al, 2000). Negative controls using irrelevant mouse immunoglobulin revealed no staining. Positive controls for p27 and cyclin E consisted of paraffin sections of breast cancer cells (Catzavelos et al, 1997; Porter et al, 1997). Tumour nuclei that showed dark brown granular staining with anti-Ki-67 were considered positive (Kamai et al, 2000). In each case, p27, cyclin E, and Ki-67 scoring was determined by examining 1000 cancer cells in 3 to 7 slide sections in each of 5 to 10 microscopic fields under ×400 magnification. The labelling index was expressed as the ratio of total labelled cells to the total number of tumour cells counted (Catzavelos et al, 1997; Loda et al, 1997; Del Pizzo et al, 1999; Kamai et al, 2000). Scoring was done by 3 of the authors independently (TK, HA and YI). The labelling index threshold was based on methods described previously (Catzavelos et al, 1997; Loda et al, 1997; Del Pizzo et al, 1999; Kamai et al, 2000). p27 scoring was classified into 2 categories of tumour cell nuclear staining; low (<50%) and high (>50%) (Catzavelos et al, 1997; Loda et al, 1997; Kamai et al, 2000), cyclin E; low (<30%) and high (>30%) (Del Pizzo et al, 1999) and Ki-67 labelling; low (<30%) and high (>30%) (Kamai et al, 2000).

### Statistical analysis

The chi-square test and Fisher's exact test were used for comparisons between p27 staining and cyclin E scoring and for prognostic variables including histological grade, pathological stage, lymph node metastasis and tumour proliferation by the Ki-67 index. p27 and cyclin E scoring and prognostic variables were analysed for disease-free and overall survival by the Cox proportional hazards model using univariate and multivariate analysis. The Kaplan–Meier method was used to estimate survival as a function of time, and survival differences were analysed by the log-rank test. *P* values less than 0.05 were considered significant. Data were analysed with commercial software.

### RESULTS

### Relationship of p27 and cyclin E protein levels with clinical characteristics

Cells showing positive reactions for anti-p27$^{Kip1}$, anti-cyclin E and MIB-1 showed predominant immunostaining in the nucleus. The normal urothelium showed a uniformly strong positive reaction with anti-p27$^{Kip1}$ and a very weakly positive reaction for MIB-1. Although the overall number and intensity of cyclin E immunoreactive cells was not as high as for p27$^{Kip1}$, normal urothelium demonstrated uniformly abundant staining with anti-cyclin E. Expression of p27, cyclin E and Ki-67 was observed in 121 of 145 patients (83.4%), 107/145 (73.8%), and all patients, respectively. Tumour cells showed heterogeneous immunostaining: moderate to negative levels of immunoreactivity for p27 and cyclin E (Figures 1 and 2) and moderate to strong levels for Ki-67. The immunohistochemical analysis in relation to tumour characteristics are summarized in Table 1. Decreased protein levels of p27 correlated with poorly differentiated grade ($P = 0.0006$), higher stage ($P = 0.0019$), lymph node involvement ($P = 0.0002$), lower cyclin E staining ($P = 0.0302$), and higher tumour proliferation ($P = 0.0306$). Decreased protein levels of cyclin E were associated with poorly differentiated grade ($P = 0.0187$), higher stage ($P = 0.0387$) and higher tumour proliferation ($P = 0.0002$), but not with lymph node disease ($P = 0.6143$). The higher Ki-67 index correlated with poorly differentiated grade ($P < 0.0001$), higher stage ($P < 0.0001$) and lymph node involvement ($P < 0.0001$).

### Relationship of p27 and cyclin E to survival

© 2001 Cancer Research Campaign 

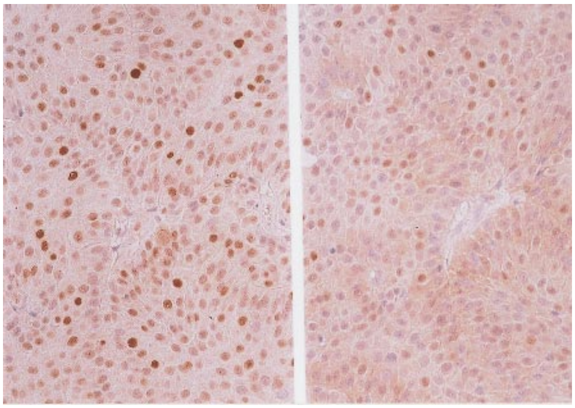

**Figure 1** Immunohistochemical staining using anti-p27$^{Kip1}$ (left panel) and anti-cyclin E (right panel) monoclonal antibody in papillary type, grade 1 and pT1 bladder tumour (× 400 magnification). Left panel shows intensely brown staining in most of the nuclei of the cancer cells, displaying high p27$^{Kip1}$ protein levels. Right panel shows weakly brown staining in many of the nuclei of the cancer cells from the same section of left panel, showing high cyclin E protein levels

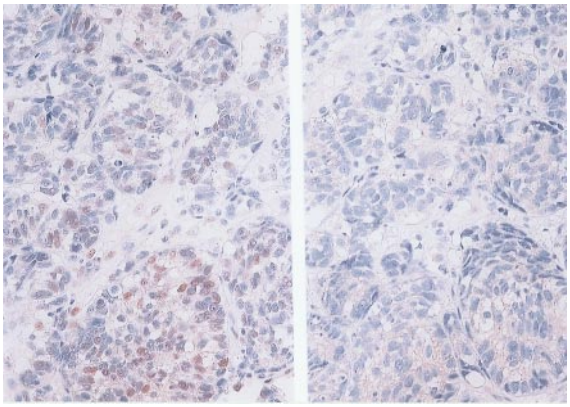

**Figure 2** Immunohistochemical staining using anti-p27$^{Kip1}$ (left panel) and anti-cyclin E (right panel) monoclonal antibody in non-papillary type, grade 3 and pT3 bladder tumour (× 400 magnification). Left panel shows very weakly brown staining in some of the nuclei of the cancer cells, diaplaying low p27$^{Kip1}$ protein levels. Right panel shows negative reaction for anti-cyclin E monoclonal antibody from the same section of left panel, displaying low cyclin E protein levels

Univariate disease-free survival analysis revealed that p27 ($P < 0.0001$, relative risk [RR] = 4.369), Ki-67 ($P < 0.0001$, RR = 3.876), stage ($P < 0.0001$, RR = 1.826), cyclin E ($P = 0.0004$, RR = 2.589), grade ($P = 0.0005$, RR = 1.878), and lymph node disease ($P = 0.0363$, RR = 2.469) had significant survival effects (Table 2). p27 and Ki-67 were independent predictors of a shortened disease-free survival ($P < 0.0001$, RR = 3.879 and $P = 0.0031$, RR = 2.914, respectively) by multivariate analysis.

With regard to overall survival, univariate analysis showed that p27 ($P < 0.0001$, RR = 12.878), cyclin E ($P < 0.0001$, RR = 6.346), Ki-67 ($P < 0.0001$, RR = 23.352), stage ($P < 0.0001$, RR = 3.484), grade ($P < 0.0001$, RR = 3.724), and lymph node involvement ($P = 0.0019$, RR = 4.624) were significant. On multivariate analysis, Ki-67 ($P = 0.0003$, RR = 12.971), cyclin E ($P = 0.0009$, RR = 7.749), stage ($P = 0.0011$, RR = 2.640), and p27 ($P = 0.0017$, RR = 10.434) were independent prognostic factors for overall survival (Table 2).

Kaplan–Meier plots of survival rates in patients with low versus high p27 staining showed that low staining of p27 correlated with shortened disease-free and overall survival ($P < 0.0001$ and $P < 0.0001$, respectively, Figure 3A, C). Low staining of cyclin E was associated with early recurrence and a lower overall survival ($P = 0.0003$, $P < 0.0001$, respectively, Figure 3B, D). The prognosis of patients with a high Ki-67 index was poor for both disease-free and overall survival in comparison to those with a low Ki-67 index ($P < 0.0001$, $P < 0.0001$, respectively, data not shown).

We examined the role of p27 and cyclin E protein levels in high and low Ki-67 index patients. In the low Ki-67 index group (89 patients) there was no difference in survival rates between high and low p27 staining or between high and low cyclin E staining (data not shown). In contrast, in the high Ki-67 index group (56 patients), patients with high p27 staining showed a better prognosis than those with low p27 staining for both disease-free and overall survival ($P = 0.0055$, $P = 0.0026$, respectively, Figure 4A, C). High cyclin E staining was associated with a favourable prognosis for overall ($P = 0.0022$) but not disease-free survival ($P = 0.0637$) in the high Ki-67 index group (Figure 4B, D). The prognosis of patients with low protein levels of both p27 and cyclin E and a high Ki-67 index was extremely poor.

### Analysis of p27 and cyclin E in superficial bladder cancer

By comparison, which of grade, p27, cyclin E and Ki-67 has a significant influence on disease-free survival in superficial tumours (Ta, T1; 86 patients), p27 and Ki-67 were statistically significant predictors by univariate ($P = 0.0057$, $P = 0.0098$, respectively) and multivariate analysis ($P = 0.0048$ and $P = 0.0178$, respectively, Table 4). Kaplan–Meier plots revealed that there was a significant correlation between the combination of low expression of p27 and a high Ki-67 index and early recurrence ($P = 0.003$, $P = 0.0063$, respectively, Figure 5B, C).

35 of 86 patients with superficial bladder tumours suffered recurrence. We classified these tumours into 2 groups in order to evaluate the roles of the alterations of expression status of p27, cyclin E, and Ki-67 in the progression of bladder tumours (Table 4). In group A (26 patients), although the superficial tumour recurred, the tumour remained at a low stage, and these patients showed a better prognosis. In group B (9 patients), the recurrent tumour finally invaded the muscle layer, and the prognosis of these patients was poor. At first operation, the Ki-67 index was higher in group B than in group A ($P = 0.0007$), but no difference was found between the groups in expression levels of p27 and cyclin E. In contrast, higher protein levels of p27 ($P = 0.0149$) and cyclin E ($P = 0.0165$) were observed in group A in comparison to group B at the second operation, while the difference previously noted for Ki-67 index was no longer apparent. The similar expression pattern of these protein levels was found in the final operation (3–7 times).

### DISCUSSION

In G1/S phase, the p16$^{INK4a}$-cyclin D1-Cdk4/6-retinoblastoma gene (Rb) pathway contributes greatly to tumour development (Hunter and Pines, 1994). In bladder cancer, deletions of p16$^{INK4a}$ (Orlow et al, 1999), low expression of Rb (Cordon-Cardo et al, 1992; Logothetis et al, 1992), and overexpression of cyclin D1 (Lee et al,

*© 2001 Cancer Research Campaign*

**Table 1** Comparison of p27 staining with grade, stage, cyclin E and Ki-67 in 145 patients

| | No. | No. of high p27 | No. of low p27 | | No. of high cyclin E | No. of low cyclin E | | No. of low Ki-67 | No. of high Ki-67 | |
|---|---|---|---|---|---|---|---|---|---|---|
| | | **79** | **66** | ***P* value** | **78** | **67** | ***P* value** | **89** | **56** | ***P* value** |
| Tumour grade | | | | | | | | | | |
| G1 | 39 | 28 | 11 | | 25 | 14 | | 32 | 7 | |
| G2 | 58 | 34 | 24 | 0.0006 | 34 | 24 | 0.0187 | 46 | 12 | < 0.0001 |
| G3 | 48 | 17 | 31 | | 19 | 29 | | 11 | 37 | |
| Pathological stage | | | | | | | | | | |
| Ta, T1 | 86 | 56 | 30 | | 53 | 33 | | 70 | 16 | |
| T2 | 26 | 11 | 15 | 0.0019 | 10 | 16 | 0.0387 | 10 | 16 | < 0.0001 |
| T3, T4 | 33 | 12 | 21 | | 15 | 18 | | 9 | 24 | |
| Lymph node | | | | | | | | | | |
| metastasis (–) | 137 | 77 | 60 | 0.002 | 73 | 64 | 0.6143 | 87 | 50 | < 0.0001 |
| metastasis (+) | 8 | 2 | 6 | | 5 | 3 | | 2 | 6 | |
| Cyclin E | | | | | | | | | | |
| high | 78 | 49 | 29 | 0.0302 | | | | | | |
| low | 67 | 30 | 37 | | | | | | | |
| Ki-67 | | | | | | | | | | |
| low | 89 | 56 | 33 | 0.0306 | 61 | 28 | 0.0002 | | | |
| high | 56 | 23 | 33 | | 17 | 39 | | | | |

© 2001 Cancer Research Campaign

**Table 2** Cox regression analysis for various potential prognostic factors in survival

| Variable | Unfavourable/ favourable characteristics | No. of patients | Analysis | Disease-free survival | | | Overall survival | | |
|---|---|---|---|---|---|---|---|---|---|
| | | | | **Relative risk** | **95% confidence interval** | ***P* value** | **Relative risk** | **95% confidence interval** | ***P* value** |
| Grade | 3/2/1 | 48/58/39 | Univariate (U) | 1.878 | 1.320–2.673 | 0.0005 | 3.724 | 1.994–6.955 | <0.0001 |
| | | | Multivariate (M) | 1.092 | 0.569–1.403 | 0.6205 | 1.158 | 0.716–1.370 | 0.2628 |
| pT | >3/2/1,a | 33/26/86 | U | 1.826 | 1.377–2.421 | <0.0001 | 3.484 | 2.210–5.462 | <0.0001 |
| | | | M | 1.295 | 0.880–1.904 | 0.1895 | 2.64 | 1.472–4.733 | 0.0011 |
| Lymph node metastasis | + /– | 8/137 | U | 2.469 | 1.059–5.755 | 0.0363 | 4.624 | 1.762–12.134 | 0.0019 |
| | | | M | 1.321 | 0.481–3.624 | 0.5893 | 2.749 | 0.754–10.195 | 0.1255 |
| Ki–67 | high/low | 56/89 | U | 3.876 | 2.280–6.590 | <0.0001 | 23.352 | 6.971–78.232 | <0.0001 |
| | | | M | 2.914 | 1.435–5.916 | 0.0031 | 12.971 | 3.232–52.061 | 0.0003 |
| p27 | low/high | 66/79 | U | 4.369 | 2.315–8.246 | <0.0001 | 12.878 | 2.581–17.748 | <0.0001 |
| | | | M | 3.879 | 2.021–7.444 | <0.0001 | 10.434 | 2.419–45.004 | 0.0017 |
| Cyclin E | low/high | 67/78 | U | 2.589 | 4.399–8.246 | 0.0004 | 6.346 | 2.572–15.663 | <0.0001 |
| | | | M | 1.77 | 0.970–3.230 | 0.0629 | 7.749 | 2.315–25.939 | 0.0009 |

*© 2001 Cancer Research Campaign*

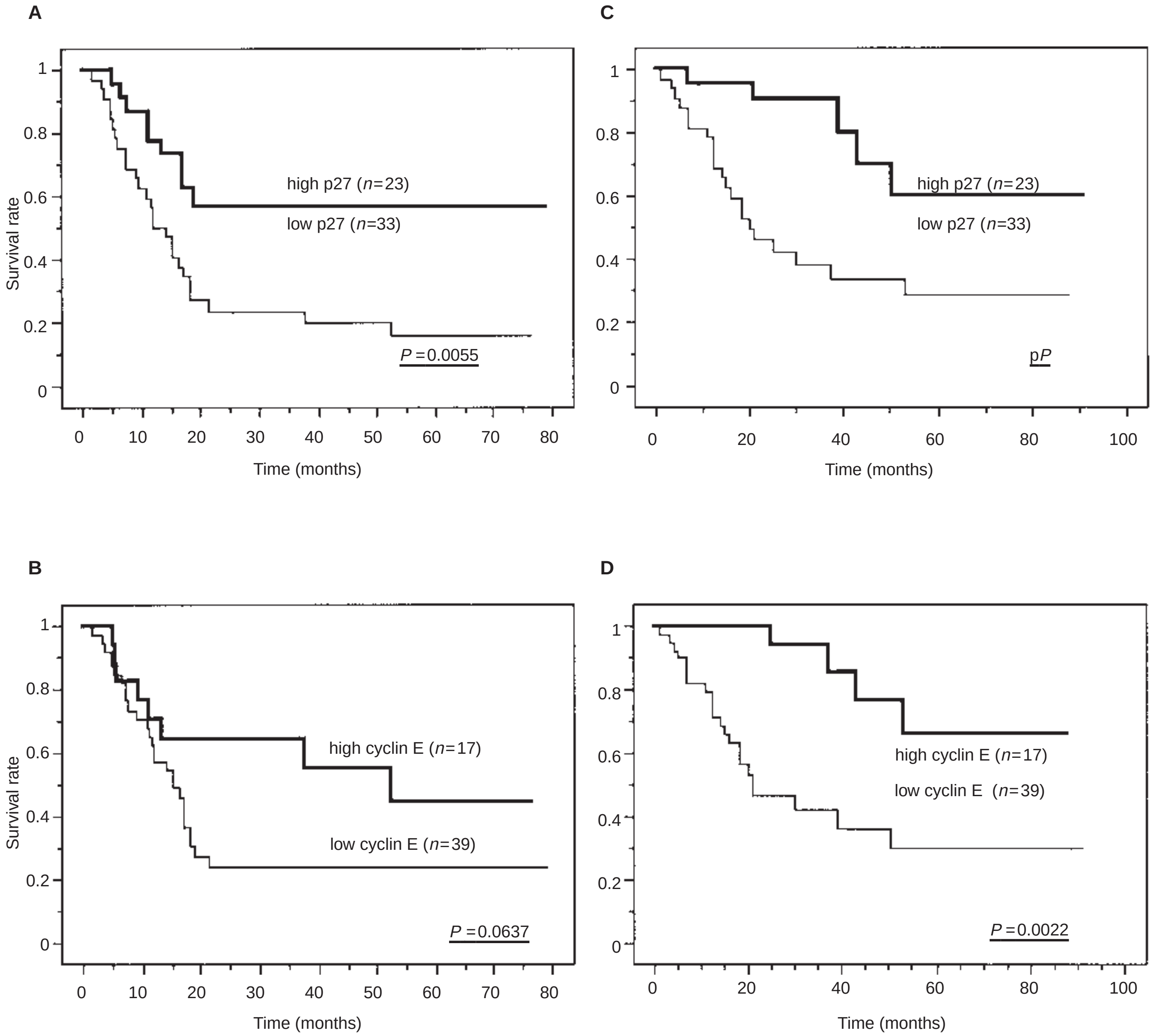

**Figure 3** Survival curve in 145 patients with bladder cancer based on the p27 (**A**; Disease-free survival curve. **C**; Overall survival curve) and cyclin E immunointensity (**B**; Disease-free survival curve. **D**; Overall survival curve). *P* value was analysed by log-rank test. The prognosis of the patients with high p27 and cyclin E staining was better than those with low p27 and cyclin E staining

1997; Shin et al, 1997) are involved in progression of these tumours. p16$^{INK4a}$, p21$^{WAF/CIP1}$, p27, cyclin D1-Cdk4/6 complex and cyclin E-Cdk2 complex are involved in the G1/S transition by regulating phosphorylation of Rb (Toyoshima and Hunter, 1994). Among these, p27 and cyclin E play a central role in the transition from late G1 to S phase (Steeg and Abrams, 1997). Therefore, it is possible to assess the role of p27 and cyclin E in tumour recurrence and progression and to define their value in predicting survival in patients with bladder cancer.

There is a significant inverse correlation of p27 protein levels with grade and stage. Low p27 protein levels are associated with poorer survival in breast (Catzavelos et al, 1997), gastric (Mori et al, 1997), prostate (Tsihlias et al, 1998), upper uninary tract (Kamai et al, 2000), and bladder cancer (Del Pizzo et al, 1999; Lee et al, 1999). In addition, low protein levels of p27 were a negative prognostic factor in lung (Esposito et al, 1997), colorectal (Loda et al, 1997), and ovarian cancer (Masciullo et al, 1999). In the present study, low protein levels of p27 were associated with poorly differentiated grade, muscle and lymph node invasion, and unfavourable prognosis. Therefore, it is likely that p27 affects differentiation pathways and acts as a tumour suppressor gene in different human tumours; evaluation of p27 protein levels may indicate the biological behaviour of human tumours.

High protein levels of cyclin E are associated with increasing tumour grade and mortality in breast cancer (Keyomarsi et al, 1995). Since p27 levels are mainly regulated by ubiquitin-mediated proteasome degradation, which is targeted by cyclin E-Cdk2 phophorylation of p27 (Vlach et al, 1997), it is likely that loss of p27 expression and high expression of cyclin E are associated with cancer progression and unfavourable prognosis. Low protein levels of p27 and increased protein levels of cyclin E correlated with shortened survival in breast cancer (Porter et al, 1997). In contrast, low staining of p27 and cyclin E correlated with poorly differentiated grade, muscle invasion, lymph node involvement and poor survival in bladder cancer patients in this and previous studies (Del Pizzo et al, 1999). There may be different

© 2001 Cancer Research Campaign

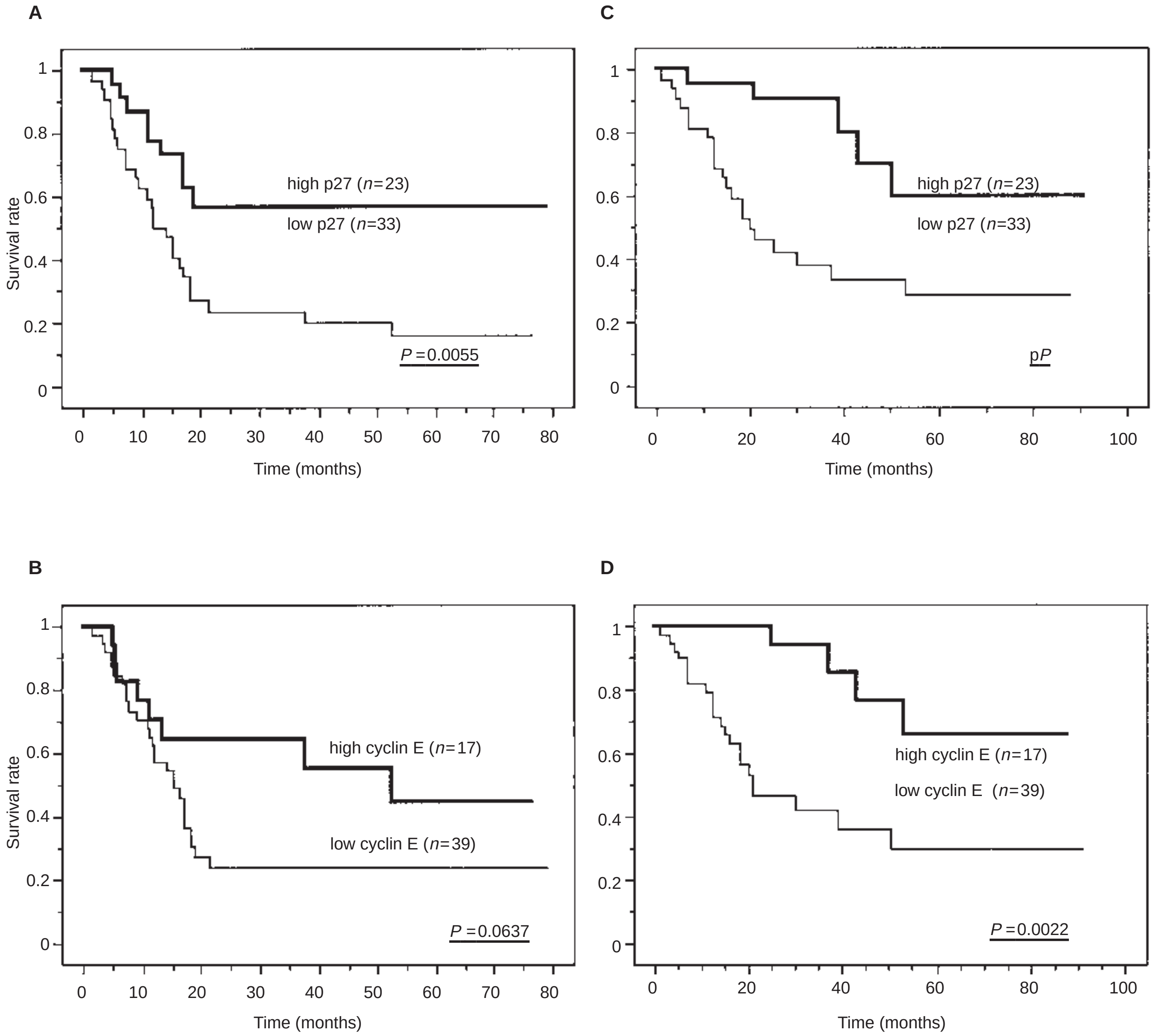

**Figure 4** Survival curve in high Ki-67 labelling index patients based on the p27 (**A**; Disease-free survival curve. **C**; Overall survival curve) and cyclin E (**B**; Disease-free survival curve. **D**; Overall survival curve) immunointensity. *P* value was analysed by log-rank test. Decreased p27 staining was associated with decreased relapse-free survival and with increased disease-specific mortality and decreased cyclin E staining was associated with increased disease-specific mortality regulatory mechanisms in expression of these genes between breast and bladder cancer. Del Pizzo et al (1999) suggested that cellular down-regulation of cyclin E may be an attempt to offset loss of p27 expression during tumour growth via a feedback inhibitory loop. In the present study, there was a positive relationship between protein levels of p27 and cyclin E and a negative relationship between p27 and cyclin E staining and Ki-67 index. Furthermore, in the high Ki-67 index group whose prognosis was poor, patients with high p27 staining showed a better prognosis than those with low p27 staining.

These data suggest that p27 inhibits bladder tumour proliferation and progression. Low cyclin E staining was also associated with shorter overall survival in the high Ki-67 index group. These findings support the data of Del Pizzo et al (1999). On the other hand, Richter et al (2000) recently reported that cyclin E expression was significantly more frequent in pT1 than pTa tumours, but protein levels significantly decreased from pT1 to pT2-4, and low cyclin E expression was associated with poor overall survival in TCC of the bladder, suggesting that cyclin E overexpression might be characteristic to a subset of bladder cancers, especially at the stage of early invasion. We similarly observed that protein levels of cyclin E were higher in pTa-1 than pT2-4 (Table 1), these decreased as cancer became invasive (Table 4).

Superficial bladder tumours (Ta, T1) frequently recur and progress to invasive disease (Loening et al, 1980; Heney et al, 1983). One problem in the evaluation of superficial bladder tumours is the tendency to underdiagnose the extent of disease. Given the problem of underdiagnosing superficial tumours, it would be interesting to evaluate the prognostic value of p27 and cyclin E protein in these tumours. In the current study a significant correlation was observed between low protein levels of p27 and decreased disease-free survival in superficial tumours, but this was not found for cyclin E (Table 3). Similar relationship between cyclin E expression and disease-free survival in superficial

*© 2001 Cancer Research Campaign*

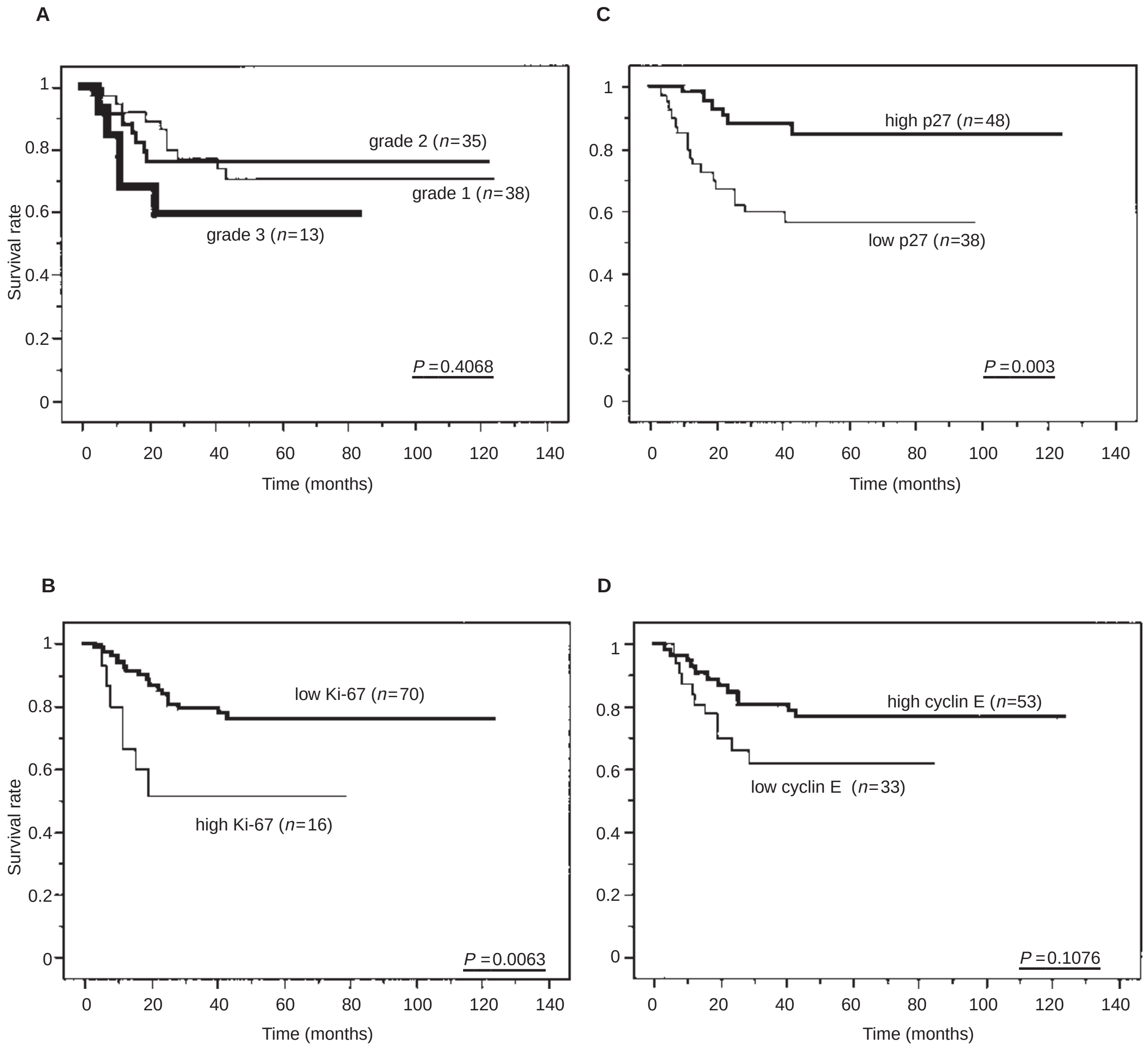

**Figure 5** Disease-free survival curve based on grade (**A**), Ki-67 (**B**), p27 (**C**) and cyclin E (**D**) in superficial tumours. *P* value was analysed by log-rank test. High Ki-67 index and low p27 staining correlated with shortened disease-free survival

**Table 3** Cox regression analysis for Grade, Ki-67, P27 and cyclin E in recurence in superficial tumours

| Variable | Unfavourable / favourable characteristics | No. of patients | Analysis | Relative risk | 95% confidence interval | *P* value |
|---|---|---|---|---|---|---|
| Grade | 3/2/ 1 | 13/35/38 | Univariate (U) | 1.288 | 0.729–2.274 | 0.384 |
| | | | Multivariate (M) | 1.16 | 0.535–1.723 | 0.96 |
| Ki-67 | high/low | 16/70 | U | 3.289 | 1.333–8.115 | 0.0098 |
| | | | M | 3.586 | 1.247–10.313 | 0.0178 |
| p27 | low/high | 38/48 | U | 3.723 | 1.467–9.448 | 0.0057 |
| | | | M | 3.898 | 1.514–10.035 | 0.0048 |
| Cyclin E | low/high | 33/53 | U | 1.941 | 0.853–4.420 | 0.1941 |
| | | | M | 1.231 | 0.514–2.951 | 0.6407 |

tumours was reported (Richter et al, 2000). Impressively, serial immunostaining over time showed that tumours with consistently high protein levels of p27 and cyclin E did not progress to invasive disease and showed a better prognosis, regardless of a high Ki-67 index (Table 4). These findings suggested that decreasing protein levels of p27 and cyclin E and increasing Ki-67 index may be associated with transformation of bladder tumour cells into more aggressive cancers. Therefore, serial examination of protein levels

*© 2001 Cancer Research Campaign*

**Table 4** Alterations of p27, cyclin E and Ki-67 in superficial bladder tumour between Group A and B

| | 1st operation | | 2nd operation | | Final operation (3–7 times) | |
|---|---|---|---|---|---|---|
| | No. of the patients | | No. of the patients | | No. of the patients | |
| | Group A 26 | Group B 9 | Group A 26 | Group B 9 | Group A 6 | Group B 9 |
| p27 | | | | | | |
| high | 17 | 5 | 21 | 3 | 6 | 2 |
| low | 9 | 4 | 4 | 6 | 0 | 7 |
| P (A vs. B) | *P* = 0.4109 | | *P* = 0.0149 | | *P* = 0.0133 | |
| cyclin E | | | | | | |
| high | 13 | 4 | 17 | 1 | 4 | 0 |
| low | 13 | 5 | 9 | 8 | 2 | 9 |
| P (A vs. B) | *P* = 0.6028 | | *P* = 0.0165 | | *P* = 0.0339 | |
| Ki-67 | | | | | | |
| low | 20 | 2 | 15 | 2 | 3 | 0 |
| high | 6 | 7 | 11 | 7 | 3 | 9 |
| P (A vs. B) | *P* = 0.0007 | | *P* = 0.0761 | | *P* = 0.1116 | |

of p27, cyclin E and Ki-67 have the potential to classify local recurrence patients into either significantly elevated or reduced risk groups. Such a classification may determine which patients would benefit from more aggressive therapy. If protein levels of p27 and cyclin E decrease with time, it may be preferable to change to more aggressive treatment methods, regardless of the Ki-67 index.

Stage, grade and lymph node metastasis are associated with survival, and treatment decisions for individual patients with bladder cancer have been based on these conventional prognostic factors. In the present study, low protein levels of p27 and cyclin E and a high Ki-67 index were associated with poorly differentiated grade, muscle invasion and unfavourable prognosis in bladder cancer. Furthermore, using multivariate analysis, p27 and Ki-67 were significant covariates for disease-free survival, and Ki-67, cyclin E, p27 and stage were factors for overall survival. These findings suggest that p27, cyclin E and Ki-67 are independent prognostic markers in bladder tumours.

We did not analyse the effect of intravesical chemotherapy or immunotherapy for superficial bladder tumours on changes in these cell cycle-regulating proteins in the current study. We demonstrated here that the decreasing of p27 and cyclin E is involved in the progression of the bladder tumour. Patients undergoing intravesical chemotherapy or immunotherapy are thought to share the same risk factors for invasive disease as those treated with transurethral resection alone (Lamm et al, 1995; Vegt et al, 1995). Fujii et al (1998) suggested that conventional pathological prognostic factors had a more important influence on the progression of superficial tumours than the choice of treatment. The role of p27 and cyclin E in treated patients will be the subject of a forthcoming study. While previous studies have not found p27 or cyclin E gene mutations in human tumours, mutations of these genes in bladder tumours are under investigation.

### REFERENCES

Catzavelos C, Bhattacharya N, Ung YC, Wilson JA, Roncari L, Sandhu C, Shaw P, Yeger H, Morava-Protzner I, Kapusta I, Franssen E, Pritchard KI and Slingerland JM (1997) Decreased levels of the cell-cycle inhibitor p27Kip1 protein; prognostic implications in promary breast cancer. *Nat Med* **3**: 227–230

Conner JP, Rpoportm F, Olsson CA, Sawczuk IS and Benson MC (1990) Long-term follow-up patients treated with methotrexate, vinblastine, doxorubicin and cisplatin (M-VAC) for transitional cell carcinoma of urinary bladder: Cause for concern. *Urology* **34**: 353–356

Cordon-Cardo C, Wartinger D, Petrylak D, Dalbagni G, Fair WR, Fuks Z and Reuter VE (1992) Altered expression of the retinoblastoma gene product: Prognostic indicator in bladder cancer. *J Natl Cancer Inst* **84**: 1251–1256

Del Pizzo JJ, Borkowski A, Jacobs SC and Kyprianou N (1999) Loss of cell cycle regulators $p27^{Kip1}$ and cyclin E in transitional cell carcinoma of the bladder correlates with tumor grade and patient survival. *Am J Pathol* **155**: 1129–1136

Esposito V, Baldi A, Luka AD, Groger AM, Loda M, Giordano GG, Caputi M, Baldi F, Pagano M and Giordano A (1997) Prognostic role of the cyclin-dependent kinase inhibitor p27 in non-small cell lung cancer. *Cancer Res* **57**: 3381–3385

Fujii Y, Fukui I, Kihara K, Tsujii T, Ishizaka K, Kageyama Y, Kawakami S and Oshima H (1998) Significance of bladder neck involvement on progression in superficial bladder cancer. *Eur Urol* **33**: 464–468

Geller NL, Sternberg CN, Penenberg D, Scher H and Yagoda A (1991) Prognostic factors for survival of patients with advanced urothelial tumors treated with methotrexate, vinblastine, doxorubicin and cisplatin chemotherapy. *Cancer* **67**: 1525–1531

Heney NM, Aiimed S, Flanagan MJ, Frable W, Corder MP, Hafermann MD and Hwkins IR for national bladder cancer collaborative group A (1983) Superficial bladder cancer: Progression and recurrence. *J Urol* **130**: 1083–1086

Hunter T and Pines J (1994) Cyclins and cancer II: cyclin D and CDK inhibitors come of age. *Cell* **79**: 573–582

Kamai T, Takagi K, Asami H, Ito Y, Arai K and Yoshida K-I (2000) Prognostic significance of $p27^{Kip1}$ and Ki-67 expression in carcinoma of the renal pelvis and ureter. *BJU Int* **86**: 14–19

Katayose Y, Kim M, Rakkar ANS, Li Z, Cowan KH and Seth P (1997) Promoting apoptosis: a novel activity associated with the cyclin-dependent kinase inhibitor p27. *Cancer Res* **57**: 5441–5445

Keyomarsi K, Conte D, Toyofuku W and Fox MP (1995) Deregulation of cyclin E in breast cancer. *Oncogene* **11**: 941–950

Kuczyk M, Machtens S, Hradil K, Schubach J, Christian W, Knüchel R, Hartmann J, Bokemeyer C, Jonas U and Serth J (1999) Predictive value of decreased $p27^{Kip1}$ protein expression for the recurrence-free and long-term survival of prostate cancer patients. *Br J Cancer* **81**: 1052–1058

Lamm DL, Riggs DR, Traynelis CL, and Nseyo UO (1995) Apparent failure of current intravesical chemotherapy propphylaxis to influence the long-term course of superficial transitional cell carcinoma of the bladder. *J Urol* **153**: 1444–1450

Lee CCR, Yamamoto S, Morimura K, Wanibuchi H, Nishisaka N, Ikemoto S, Nakatani T, Wada S, Kishimoto T and Fukushima S (1997) Significance of cyclin D1 overexpression in transitional cell carcinomas of the urinary bladder and its correlation with histopathologic features. *Cancer* **79**: 780–789

Lee CCR, Ichihara T, Yamamoto S, Wanibuchi H, Sugimura K, Wada S, Kishimoto T and Fukushima S (1999) Reduced expression of the CDK inhibitor $p27^{Kip1}$ in rat two-stage bladder carcinogenesis and its association with expression profiles of $p21^{WAF1/Cip1}$ and p53. *Carcinogenesis* **20**: 1697–1708

 *© 2001 Cancer Research Campaign*

Loda M, Cukor B, Tam SW, Lavin P, Fiorentino M, Draetta GF, Jessup Jm and Pagano M (1997) Increased proteasome-dependent degradation of the cyclin-dependent kinase inhibitor p27 in aggressive colorectal carcinomas. *Nat Med* **3**: 231–234

Loening S, Narayama A, Yoder L, Slymen D, Weinstein S, Penick G and Culp D (1980) Factors influencing the recurrence rate of bladder cancer. *J Urol* **123**: 29–31

Logothetis CJ, Xu H-J, Ro JY, Hu S-X, Sahin A, Ordonez N and Benedict WF (1992) Altered expression of retinoblastoma protein and known prognostic variables in locally advanced bladder cancer. *J Natl Cancer Inst* **84**: 1256–1261

Masciullo U, Sgambato A, Pacilio C, Pucci B, Ferrandina G, Palazzo J, Carbone A, Cittadini A, Mancuso S, Scambia G and Giordano A (1999) Frequent loss of expression of the cyclin- dependent kinase inhibitor p27 in ovarian cancer. *Cancer Res* **59**: 3790–3794

Mori M, Mimori K, Shiraishi T, Tanaka S, Ueo H, Sugimachi K and Akiyoshi T (1997) p27 expression and gastric carcinoma. *Nat Med* **3**: 593

Orlow I, LaRue H, Osman I, Lacombe L, Moore L, Rabbani F, Meyer F, Fradet Y and Cordon-Cardo C (1999) Deletions of the INK4A gene in superficial bladder tumors. Association with recurrence. *Am J Pathol* **155**: 105–113

Porter PL, Malone KE, Heagerty PJ, Alexander GM, Gatti LA, Firpo EJ, Daling JR and Roberts JM (1997) Expression of cell- cycle regulators p27Kip1 and cyclin E, alone and in combination, correlates with survival in young breast cancer patients. *Nat Med* **3**: 222–225

Rakkar ANS, Li Z, Katayose Y, Kim M, Cowan KH and Seth P (1998) Adenoviral expression of the cyclin-dependent kinase inhibitor p27$^{Kip1}$: a strategy for breast cancer gene therapy. *J Natl Cancer Inst* **90**: 1836–1838

Richter J, Wagner U, Kononen J, Fijan A, Bruderer J, Schmid U, Ackermann D, Maurer R, Alund G, Knonagel H, Rist M, Wilber K, Anabitarte M, Hering F, Hardmeier T, Schonenberger A, Flury R, Jager P, Fehr JL, Schraml P, Moch H, Mihatsch MJ, Gasser T, Kallioniemi OP and Sauter G (2000) High-throughput tissue microarray analysis of cyclin E gene amplification and overexpression in urinary bladder cancer. *Am J Pathol* **157**: 787–794

Sgambato A, Migaldi M, Faraglia B, Garagnani L, Romano G, De Gaetani C, Ferrari P, Cpelli G, Trentini GP and Cittadini A (1999) Loss of p27$^{Kip1}$ expression correlates with tumor grade and with rduced disease-free survival in primary superficial bladder cancers. *Cancer Res* **59**: 3245–3250

Shin KY, Kong G, Kim WS, Lee TY, Woo YN and Lee JD (1997) Overexpression of cyclin D1 correlates with early recurrence in superficial bladder cancer. *Br J Cancer* **75**: 1786–1792

Sobin LH, editors (1997) International union against cancer. *In* TNM classification of malignant tumours, 5th ed. Geneva: UICC

Steeg PS and Abrams JS (1997) Cancer prognostics: past, present and p27. *Nat Med* **3**: 152–154

Sternberg CN, Yagoda A, Scher HI, Watson RC, Ahmed T, Weiselberg LR, Geller N, Hollander PS, Herr HW, Sogani PC, Morse MJ and Whitmore WF (1985) Preliminary results of M-VAC (methotrexate, vinblastine, doxorubicin and cisplatin) for transitional cell carcinoma of the urothelium. *J Urol* **133**: 403–407

Toyoshima H and Hunter T (1994) p27, a novel inhibitor of G1 cyclin-cdk protein kinase activity, is related to p21. *Cell* **78**: 67–74

Tsihlias J, Kapusta L, Deboer G, Morava-Protzner I, Zbieranowski I, Bhattacharya N, Catzavelos GC, Klotz LH and Slingerland JM (1998) Loss of cyclin-dependent kinase inhibitor p27$^{Kip1}$ is a novel prognostic factor in localized human prostate adenocarcinoma. *Cancer Res* **58**: 542–548

Vegt PDJ, Debruynr FMJ, and van der Meijden APM (1995) Bacillus Calmette-Guerin in superficial bladder cancer: Consensus and controversies. *Eur Urol* **27**: 89–95

Vlach J, Hennecke S and Amati B (1997) Phosphorylation-dependent degradation of the cyclin-dependent kinase inhibitor p27. *EMBO J* **16**: 5334–5344

Yang RM, Naitoh J, Murphy M, Wang H-J, Phillipson J, Dekernion JB, Loda M and Reiter RE (1998) Low p27 expression predicts poor disease-free survival in patients with prostate cancer. *J Urol* **159**: 941–945

© 2001 Cancer Research Campaign

## References

[PDF_00628] Catzavelos C., Bhattacharya N., Ung Y. C., Wilson J. A., Roncari L., Sandhu C., Shaw P., Yeger H., Morava-Protzner I., Kapusta L. (1997). Decreased levels of the cell-cycle inhibitor p27Kip1 protein: prognostic implications in primary breast cancer.. Nat Med.

[PDF_00632] Connor J. P., Olsson C. A., Benson M. C., Rapoport F., Sawczuk I. S. (1989). Long-term follow-up in patients treated with methotrexate, vinblastine, doxorubicin, and cisplatin (M-VAC) for transitional cell carcinoma of urinary bladder: cause for concern.. Urology.

[PDF_00636] Cordon-Cardo C., Wartinger D., Petrylak D., Dalbagni G., Fair W. R., Fuks Z., Reuter V. E. (1992). Altered expression of the retinoblastoma gene product: prognostic indicator in bladder cancer.. J Natl Cancer Inst.

[PDF_00639] Del Pizzo J. J., Borkowski A., Jacobs S. C., Kyprianou N. (1999). Loss of cell cycle regulators p27(Kip1) and cyclin E in transitional cell carcinoma of the bladder correlates with tumor grade and patient survival.. Am J Pathol.

[PDF_00643] Esposito V., Baldi A., De Luca A., Groger A. M., Loda M., Giordano G. G., Caputi M., Baldi F., Pagano M., Giordano A. (1997). Prognostic role of the cyclin-dependent kinase inhibitor p27 in non-small cell lung cancer.. Cancer Res.

[PDF_00646] Fujii Y., Fukui I., Kihara K., Tsujii T., Ishizaka K., Kageyama Y., Kawakami S., Oshima H. (1998). Significance of bladder neck involvement on progression in superficial bladder cancer.. Eur Urol.

[PDF_00649] Geller N. L., Sternberg C. N., Penenberg D., Scher H., Yagoda A. (1991). Prognostic factors for survival of patients with advanced urothelial tumors treated with methotrexate, vinblastine, doxorubicin, and cisplatin chemotherapy.. Cancer.

[PDF_00653] Heney N. M., Ahmed S., Flanagan M. J., Frable W., Corder M. P., Hafermann M. D., Hawkins I. R. (1983). Superficial bladder cancer: progression and recurrence.. J Urol.

[PDF_00656] Hunter T., Pines J. (1994). Cyclins and cancer. II: Cyclin D and CDK inhibitors come of age.. Cell.

[PDF_00658] Kamai T., Takagi K., Asami H., Ito Y., Arai K., Yoshida K. I. (2000). Prognostic significance of p27Kip1 and Ki-67 expression in carcinoma of the renal pelvis and ureter.. BJU Int.

[PDF_00662] Katayose Y., Kim M., Rakkar A. N., Li Z., Cowan K. H., Seth P. (1997). Promoting apoptosis: a novel activity associated with the cyclin-dependent kinase inhibitor p27.. Cancer Res.

[PDF_00665] Keyomarsi K., Conte D., Toyofuku W., Fox M. P. (1995). Deregulation of cyclin E in breast cancer.. Oncogene.

[PDF_00667] Kuczyk M., Machtens S., Hradil K., Schubach J., Christian W., Knüchel R., Hartmann J., Bokemeyer C., Jonas U., Serth J. (1999). Predictive value of decreased p27Kip1 protein expression for the recurrence-free and long-term survival of prostate cancer patients.. Br J Cancer.

[PDF_00671] Lamm D. L., Riggs D. R., Traynelis C. L., Nseyo U. O. (1995). Apparent failure of current intravesical chemotherapy prophylaxis to influence the long-term course of superficial transitional cell carcinoma of the bladder.. J Urol.

[PDF_00679] Lee C. C., Ichihara T., Yamamoto S., Wanibuchi H., Sugimura K., Wada S., Kishimoto T., Fukushima S. (1999). Reduced expression of the CDK inhibitor p27(KIP1) in rat two-stage bladder carcinogenesis and its association with expression profiles of p21(WAF1/Cip1) and p53.. Carcinogenesis.

[PDF_00675] Lee C. C., Yamamoto S., Morimura K., Wanibuchi H., Nishisaka N., Ikemoto S., Nakatani T., Wada S., Kishimoto T., Fukushima S. (1997). Significance of cyclin D1 overexpression in transitional cell carcinomas of the urinary bladder and its correlation with histopathologic features.. Cancer.

[PDF_00706] Loda M., Cukor B., Tam S. W., Lavin P., Fiorentino M., Draetta G. F., Jessup J. M., Pagano M. (1997). Increased proteasome-dependent degradation of the cyclin-dependent kinase inhibitor p27 in aggressive colorectal carcinomas.. Nat Med.

[PDF_00709] Loening S., Narayana A., Yoder L., Slymen D., Weinstein S., Penick G., Culp D. (1980). Factors influencing the recurrence rate of bladder cancer.. J Urol.

[PDF_00712] Logothetis C. J., Xu H. J., Ro J. Y., Hu S. X., Sahin A., Ordonez N., Benedict W. F. (1992). Altered expression of retinoblastoma protein and known prognostic variables in locally advanced bladder cancer.. J Natl Cancer Inst.

[PDF_00715] Masciullo V., Sgambato A., Pacilio C., Pucci B., Ferrandina G., Palazzo J., Carbone A., Cittadini A., Mancuso S., Scambia G. (1999). Frequent loss of expression of the cyclin-dependent kinase inhibitor p27 in epithelial ovarian cancer.. Cancer Res.

[PDF_00719] Mori M., Mimori K., Shiraishi T., Tanaka S., Ueo H., Sugimachi K., Akiyoshi T. (1997). p27 expression and gastric carcinoma.. Nat Med.

[PDF_00721] Orlow I., LaRue H., Osman I., Lacombe L., Moore L., Rabbani F., Meyer F., Fradet Y., Cordon-Cardo C. (1999). Deletions of the INK4A gene in superficial bladder tumors. Association with recurrence.. Am J Pathol.

[PDF_00724] Porter P. L., Malone K. E., Heagerty P. J., Alexander G. M., Gatti L. A., Firpo E. J., Daling J. R., Roberts J. M. (1997). Expression of cell-cycle regulators p27Kip1 and cyclin E, alone and in combination, correlate with survival in young breast cancer patients.. Nat Med.

[PDF_00728] Rakkar A. N., Li Z., Katayose Y., Kim M., Cowan K. H., Seth P. (1998). Adenoviral expression of the cyclin-dependent kinase inhibitor p27Kip1: a strategy for breast cancer gene therapy.. J Natl Cancer Inst.

[PDF_00732] Richter J., Wagner U., Kononen J., Fijan A., Bruderer J., Schmid U., Ackermann D., Maurer R., Alund G., Knönagel H. (2000). High-throughput tissue microarray analysis of cyclin E gene amplification and overexpression in urinary bladder cancer.. Am J Pathol.

[PDF_00738] Sgambato A., Migaldi M., Faraglia B., Garagnani L., Romano G., De Gaetani C., Ferrari P., Capelli G., Trentini G. P., Cittadini A. (1999). Loss of P27Kip1 expression correlates with tumor grade and with reduced disease-free survival in primary superficial bladder cancers.. Cancer Res.

[PDF_00743] Shin K. Y., Kong G., Kim W. S., Lee T. Y., Woo Y. N., Lee J. D. (1997). Overexpression of cyclin D1 correlates with early recurrence in superficial bladder cancers.. Br J Cancer.

[PDF_00748] Steeg P. S., Abrams J. S. (1997). Cancer prognostics: past, present and p27.. Nat Med.

[PDF_00750] Sternberg C. N., Yagoda A., Scher H. I., Watson R. C., Ahmed T., Weiselberg L. R., Geller N., Hollander P. S., Herr H. W., Sogani P. C. (1985). Preliminary results of M-VAC (methotrexate, vinblastine, doxorubicin and cisplatin) for transitional cell carcinoma of the urothelium.. J Urol.

[PDF_00755] Toyoshima H., Hunter T. (1994). p27, a novel inhibitor of G1 cyclin-Cdk protein kinase activity, is related to p21.. Cell.

[PDF_00757] Tsihlias J., Kapusta L. R., DeBoer G., Morava-Protzner I., Zbieranowski I., Bhattacharya N., Catzavelos G. C., Klotz L. H., Slingerland J. M. (1998). Loss of cyclin-dependent kinase inhibitor p27Kip1 is a novel prognostic factor in localized human prostate adenocarcinoma.. Cancer Res.

[PDF_00763] Vegt P. D., Debruyne F. M., van der Meijden A. P. (1995). Bacillus Calmette-Guérin in superficial bladder cancer: consensus and controversies.. Eur Urol.

[PDF_00766] Vlach J., Hennecke S., Amati B. (1997). Phosphorylation-dependent degradation of the cyclin-dependent kinase inhibitor p27.. EMBO J.

[PDF_00769] Yang R. M., Naitoh J., Murphy M., Wang H. J., Phillipson J., deKernion J. B., Loda M., Reiter R. E. (1998). Low p27 expression predicts poor disease-free survival in patients with prostate cancer.. J Urol.

